# Verification, analytical validation and clinical validation (V3) of wearable dosimeters and light loggers

**DOI:** 10.1177/20552076221144858

**Published:** 2022-12-25

**Authors:** Manuel Spitschan, Karin Smolders, Benjamin Vandendriessche, Brinnae Bent, Jessie P Bakker, Isaac R Rodriguez-Chavez, Céline Vetter

**Affiliations:** 1 Translational Sensory & Circadian Neuroscience, Max Planck Institute for Biological Cybernetics, Tübingen, Germany; 2 Chronobiology & Health, TUM Department of Sport and Health Sciences (TUM SG), 9184 Technical University of Munich, Munich, Germany; 3 TUM Institute for Advanced Study (TUM-IAS), 9184 Technical University of Munich, Garching, Germany; 4 Human-Technology Interaction Group, 3169 Eindhoven University of Technology, Eindhoven, The Netherlands; 5 Byteflies, Antwerp, Belgium; 6 Department of Electrical, Computer, and Systems Engineering, 2546 Case Western Reserve University, Cleveland, OH, USA; 7 Edge Analytics Inc., Campbell, CA, USA; 8 Signifier Medical Technologies, Needham, MA, USA; 9 Center for Decentralized Clinical Trials and Digital Medicine, ICON plc, Blue Bell, PA, USA; 10 Department of Integrative Physiology, University of Colorado Boulder, Boulder, CO, USA

**Keywords:** wearables < personalized medicine, light exposure, circadian rhythms, sleep, neuroscience < medicine, neurology < medicine, prevention < disease, health < general, electronic < general

## Abstract

**Background:**

Light exposure is an important driver and modulator of human physiology, behavior and overall health, including the biological clock, sleep-wake cycles, mood and alertness. Light can also be used as a directed intervention, e.g., in the form of light therapy in seasonal affective disorder (SAD), jetlag prevention and treatment, or to treat circadian disorders. Recently, a system of quantities and units related to the physiological effects of light was standardized by the International Commission on Illumination (CIE S 026/E:2018). At the same time, biometric monitoring technologies (BioMeTs) to capture personalized light exposure were developed. However, because there are currently no standard approaches to evaluate the digital dosimeters, the need to provide a firm framework for the characterization, calibration, and reporting for these digital sensors is urgent.

**Objective:**

This article provides such a framework by applying the principles of *verification*, *analytic validation* and *clinical validation* (V3) as a state-of-the-art approach for tools and standards in digital medicine to light dosimetry.

**Results:**

This article describes opportunities for the use of digital dosimeters for basic research, for monitoring light exposure, and for measuring adherence in both clinical and non-clinical populations to light-based interventions in clinical trials.

## Introduction

### The importance of light for human health and well-being

Exposure to light has a powerful impact on human health and well-being.^1,2^ This impact is mediated by a range of physiological responses to ocular light exposure. By illuminating the world around us, light enables us to see and perceive the world, contributing to visual performance, inducing visual experiences and affecting visual comfort. Beyond vision, light is crucial for everyday function: It is the main time cue for the circadian system, which for example regulates the phase and amplitude of an individual's daily sleep-wake pattern^3^ but also, basic physiological functions such as immune function^4^ and metabolism.^5^ In addition, a well-functioning circadian system is important for healthy sleep and optimal functioning during wakefulness. Disturbances in sleep-wake patterns due to the absence of a strong time cue or an ill-timed signal for the circadian system can result in a lack of energy, cognitive deficits, reduced self-control and a negative mood.^6–8^ Light can also induce more instantaneous changes in alertness, mood, and performance,^9–12^ independently of the crucial role of light in sleep and circadian functioning. Through these acute effects, light can – depending on its strength and timing – benefit or challenge our daytime functioning and overall physiology. Recently, consensus-based recommendations for healthy light exposure patterns were put forward by an international group of experts^13^ – further highlighting the currently under-recognized potential of light for human health. Building regulations and recommendations have been developed to address specifically the requirements of illumination vis-à-vis the physiological effects of light^14–16^ (reviewed by Ref.^17^).

### Light exposure needs to be measured in a personalized fashion

With light playing such an important role in controlling and modifying various aspects of our physiology, behavior, and long-term health, the measurement of light is of great importance. The measurement of light – called *optical radiation metrology* – is a very mature field, with many commercially available high-quality instruments for stationary measurements, such as those of a specific light source. However, humans are not stationary, moving around in and between different environments that are illuminated by diverse light sources, including electric light, daylight and self-emitting displays such as computer monitors or smartphones, which are themselves not constant due to weather conditions and daylight availability.^18,19^ Rather than measuring light in a location-specific or source-centric view, there is therefore the need to characterize light exposure in an personalized fashion.^20^ Importantly, such characterization is not limited to the physiological “non-visual” responses to light, but can equally include the characterization of the “visual diet”, i.e., which types of colors and illuminances people are exposed to.^21^

Various research groups and commercial manufacturers have developed measurement or sensor solutions that are person-based, as they are worn on different parts of the body (e.g., as a brooch, wrist-worn watch-like device, on a pair of glasses, etc.), and allow for prolonged monitoring of light exposure in free-living conditions.^21–30^

Critical factors influencing the measurements include the position of the sensor (and e.g., the possibility of the sensor being covered by clothing such as sleeves or scarves), the vulnerability of the sensor (e.g., waterproof grading, ruggedness, position on the body and variability of that position^31–34^), battery life, primary function of the sensor (e.g., is it a primary actigraphy monitor, optical monitor, or a light sensor designed to capture different dimensions of environmental light exposure), and acquisition-related parameters such as the recording interval. Systematic in-laboratory comparisons of the optical performance of light-logging sensors show large differences in their ability to capture biologically relevant light in commonly used sensors.^25,34–40^

A recent comprehensive review on light-dosimetry studies showed that in the published literature, there is a large variability of parameters used in these studies.^20^ The authors found a total of 25 unique device types used in the literature. In more than 70% of the surveyed studies, there was no mention of the dosimeter's calibration. Device placement was found to vary widely between studies, with the wrist location being the most commonly used one (64% of the surveyed studies). The recording interval also varied, with 1-minute intervals being the most commonly used one (49% of the surveyed studies). Given this diversity, it is clear that a framework is needed to understand the choice of different parameters systematically.

### Typical workflow for measuring personalized light exposure

**Figure 1.**
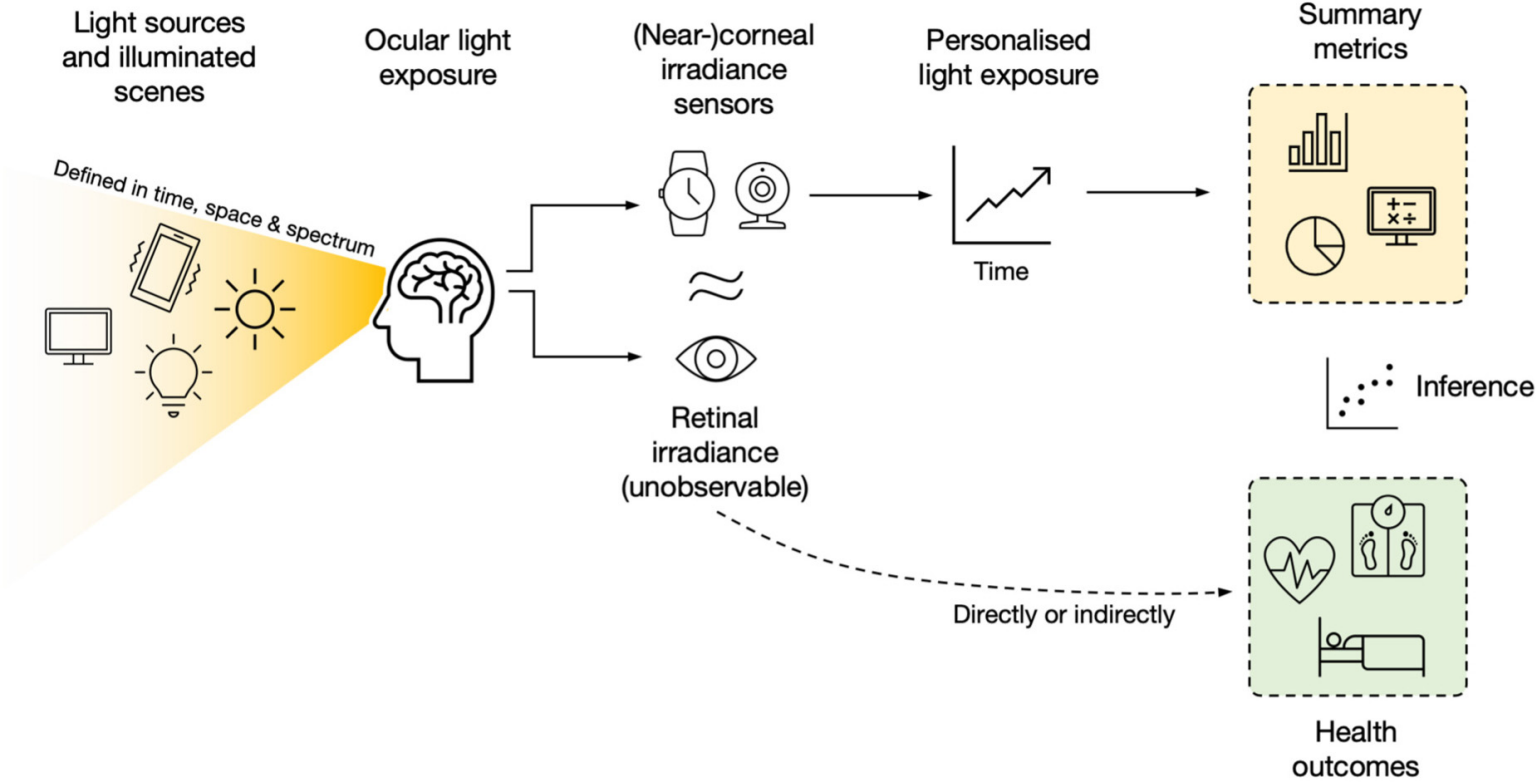
Overview of the light-dosimetry and light-logging pipeline.

Figure 1 visualizes the general workflow of personalized light-dosimetry measurements. The world contains scenes that are illuminated by different light sources, including daylight, electric light of various types, and mixtures thereof. Scenes contain materials that reflect light in potentially spectrally biased ways. Consequently, the light reaching the eye – ocular light exposure – is a complex combination of light sources, illumination geometry, and materials, all of which may be changing over time. Ocular light exposure itself is modified by dynamic parameters, such as head and eye movements, and individual-level parameters, such as facial features.

The quantity that drives the physiological effects of light – and the associated health outcomes – is the irradiance reaching the retina in the back of the eye, after passing through the pupil and being filtered by the ocular media. As retinal irradiance is not measurable by external tools – only the retina can “measure” it^41^ – proxy measurements using irradiance sensors in, near or outside of the corneal plane are used to approximate retinal irradiance. Most commonly, wrist-worn devices with build-in light-logging facilities are used (64% of the surveyed studies in Ref.^20^). When worn continuously, these measurement sensors yield time series of personalized light exposure. For research purposes, these are then typically summarized using level- (e.g., mean or median) or time-based (e.g., time above threshold) metrics and related to health-related outcomes by way of statistical association.

Of course, at the start of each investigation using personalized light exposure measurements is a research question. Different research questions will prioritize different methodological choices and outcomes: Some research questions may not require a measurement device to be accurate. In the following, we approach the question of light dosimetry from a device-dependent perspective, rather than considering the many idiosyncrasies that could arise in different research questions.

### Applications of measuring personalized light exposure in medicine and health

There are broadly four distinct modes of using light dosimetry for investigating health-related outcomes:

### Monitoring light exposure in observational studies

Light exposure patterns among free-living subjects have been captured in a number of studies. This includes research primarily describing and characterizing the patterns of light exposure across seasons and in different populations,^26,42–47^ as well as studies associating light exposure and health-related outcomes.^48–58^ The biological rationale for hypothesized associations between light exposure and health-related outcomes is clearly established through in-laboratory findings in humans and animals.^2,13^

### Monitoring light exposure as an outcome or mediator in clinical trials

In some cases, light exposure can represent the primary or secondary outcome of a specific clinical trial, such as in studies attempting to modify lifestyle to alter light exposure and visual behavior in myopia control (e.g., Ref.^30^), or studies aimed to optimize light exposure in order to support sleep and/or daytime functioning (e.g., Ref.^59^). In these studies, personalized light exposure is the study outcome, and therefore measurements need to be sensitive enough to capture change in light patterns in response to the trial intervention. Similarly, studies that focus on lifestyle interventions to improve sleep might affect light exposure (e.g., an increase in physical activity outdoors might be the behavioral intervention), and the potential increase in light exposure may affect sleep. Measuring the direct and indirect effects of an intervention, in turn, informs our causal models of disease, and enables us to maximize impact in future study designs.

### Monitoring light exposure to confirm compliance in clinical trials and in-laboratory studies

Light is used as an intervention in a range of disorders and syndromes, including depression,^60^ postpartum depression,^61^ cancer,^62–64^ dementia^65^ and delayed phase sleep disorder.^66^ Typically, these interventions involve the application and exposure to bright light sources in an ambulatory or stationary setting. For participants and patients who can freely move around, light dosimetry can therefore be used for confirming the actual effective dose (and thereby adherence to the intervention). Similarly, light exposure can be measured prior to laboratory sessions to estimate the effective dose in in-laboratory interventional studies.^67^

### Monitoring light exposure for diagnostic purposes

There are wide reaching consequences of environmental light exposure,^68^ some of which have been the focus of large-scale epidemiological studies where light exposure is measured using either stationary sensors (e.g., to assess light at night in a bedroom) or at an ecological level (e.g., using satellite data for certain geographical location, often averaged across time). These studies have provided some evidence that exposure to ambient light at night is associated with an increased risk of diabetes,^69^ breast cancer,^70–72^ depression^73^ and obesity.^50,74–77^ Effect sizes are generally small, and replication of longitudinal studies using incident disease outcomes is scarce. In addition, biological research clearly shows that the neural responses caused by light exposure are tracked and summed over time and that physiological responses depend on prior light history,^78–81^ so non-personalized light exposure measurements, especially over a short timeframe, are likely to misestimate the true impact of light exposure on long-term health.

### The V3 framework: verification, analytical validation and clinical validation

**Figure 2.**
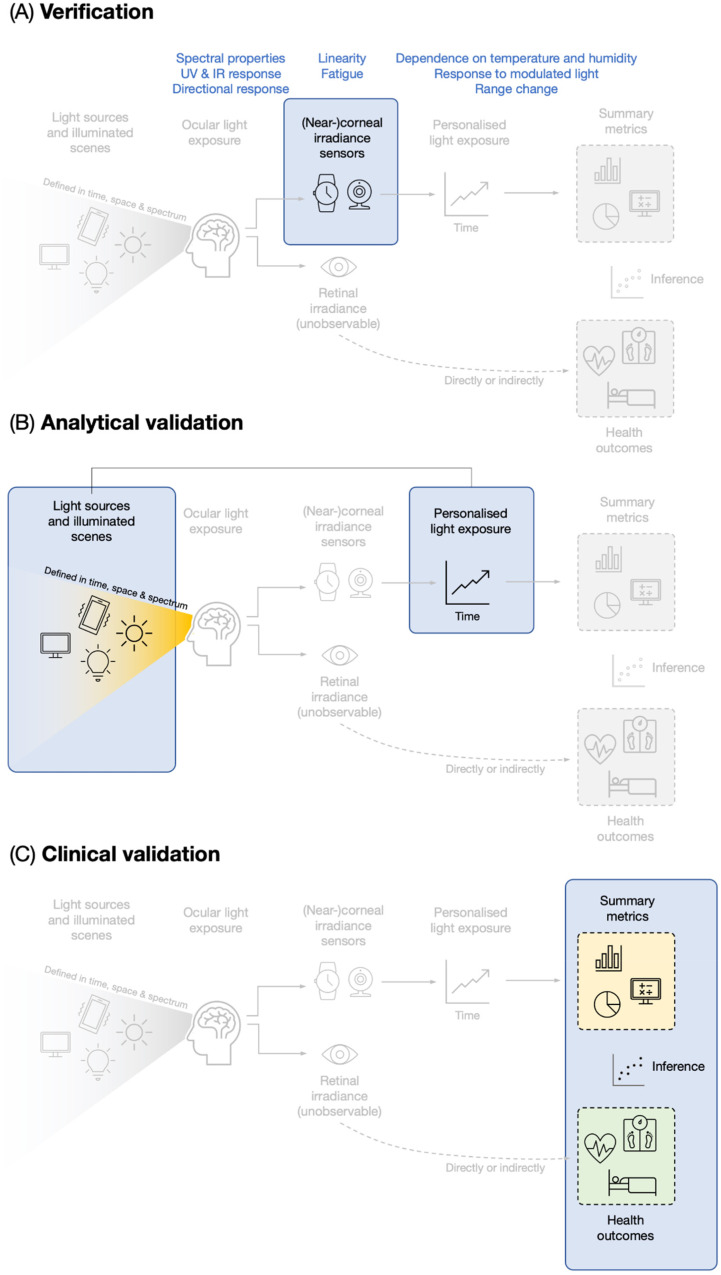
Mapping the V3 model onto light dosimetry and light logging.

The main goal of this article is to outline the steps required to advance personalized light exposure assessment to become fit-for-purpose as a medical intervention and health-related outcome and predictor in (digital) clinical trials. To do so, we have adopted the V3 framework (Box 1) which was developed to enhance reliable and robust usage of biometric monitoring technologies (or BioMeTs) in clinical research.^82^ This framework includes verification (Figure 2A), analytical validation (Figure 2B) and clinical validation, which we apply to the use of light dosimeters (Figure 2).

Box 1: V3 framework

The V3 evaluation framework^82^ is a three-component process intended to provide guidance on evaluating biometric monitoring technologies (BioMeTs) as fit-for-purpose. BioMeTs are connected digital medicine products that process data captured by mobile sensors using algorithms to generate measures of behavioral and/or physiological function.

The framework includes (1) verification, (2) analytical validation, and (3) clinical validation, described in turn below. The V3 evaluation framework was developed by the Digital Medicine Society to synthesize the various definitions and standards that had already been in use within engineering, regulatory, and clinical fields.

**Verification** refers to the evaluation of a sensor(s) within a BioMeTs and the sample-level data it generates. Verification is typically performed during bench top testing and aims to determine whether the sensor output data captures the physical property (light, in this case) within an acceptable level of accuracy, precision, consistency, and uniformity for the intended purpose.

**Analytical validation** is the evaluation of the performance of the algorithm that processes the sample-level data captured by a sensor, and its ability to measure, detect, or predict physiological or behavioral metrics. Analytical validation is typically conducted in research or clinical studies with human participants.

**Clinical validation** refers to the evaluation of whether the output of the algorithm acceptably identifies, measures, or predicts a meaningful clinical or functional experience in the stated context of use and in the specified population. Clinical validation typically occurs in the environment where the digital tool will be used. Clinical validation is generally not ‘achieved’ with a single clinical study; instead, clinical validation typically involves the evaluation of a body of work conducted over several years.

Put simply, the V3 process aims to answer the following questions in turn:

- To what extent does the output of the sensor measure the physical concept of interest within acceptable performance criteria (i.e., is the sensor contained within the device/product adequately capturing light exposure)?
- To what extent does the output of the algorithm capture the relevant behavioral or physiological phenomenon it claims to measure (e.g., is the output data an accurate measurement of physiologically relevant light exposure)?
- Is the behavioral or physiological phenomenon clinically relevant for the population of interest and context of use (e.g., does circadian phase estimated with light sensor data predict a relevant downstream health consequence)?

### Verification of light dosimeters

The goal of verification is to determine if the light sensor is accurate with respect to a reference standard, precise (intra-sensor assessment over short time periods), consistent (intra-sensor assessment over longer time periods), and uniform (inter-sensor assessment). In the case of light sensors, the International Commission on Illumination (CIE), the standards body for light-related quantities, has yet to release a standard characterizing the properties of light sensors for personalized light exposure. For illuminance meters, the international standard ISO/CIE 19476:2014 – Characterization of the performance of illuminance meters and luminance meters^83^ considers a range of properties when determining the performance of illuminance meters. Though this standard focuses specifically on measurements of photopic illuminance, its general principles of calibration are useful and can be applied to light sensors capturing biologically relevant light exposure (Figure 2A). We consider here the most important points:

- **Spectral properties**: Sensors have specific spectral sensitivity profiles, i.e., they sense light in a wavelength-dependent manner. The spectral sensitivity properties of sensors need to be characterized. The gold standard for such a characterization is to measure the response of the sensor to different monochromatic or narrowband lights, to characterize the per-wavelength response of the sensor. This requires a monochromator or a digitally tunable light source that generates single-wavelength light. For a sensor to be useful in the quantification of physiological responses, it is necessary to also characterize the extent to which the α-opic spectral sensitivities (see Box 2) can be reconstructed from the individual channel responses.
- **UV and IR response**: It is conceivable that sensor channels respond to light outside of the visible range, namely in the ultraviolet (UV) and infrared (IR) range. If sensor channels also respond to non-visible light, scenarios with significant UV or IR components may lead to an overestimation of the captured quantities in the visible range. As a consequence, if there is substantial UV or IR radiation that is picked up by the sensor channels, the measurements are inaccurate. To ensure that the sensors only register light that they purport to measure, it is important to confirm this.
- **Directional response**: Irradiance sensors capture light in a spatially integrated fashion, by integrating over the hemisphere in a cosine-corrected fashion. Rather than capturing just a single point or area in front of the sensor, it weights light coming from the center most strongly, with light coming from more acute angles carrying less weight. Whether or not a sensor and its measurement optics follow a cosine response must be carefully characterized.

Box 2: Metrology for the visual and non-visual responses of light

When discussing light, it is important to understand that the measurement and characterization of light is intimately linked to human physiology and behavior. Illuminance [lux] and luminance [cd/m^2^] are photometric quantities that imply a human observer, as they represented the spectral irradiance or radiance as weighted by the photopic luminous efficiency function *V*(*λ*). *V*(*λ*) was derived in 1924 by the International Commission on Illumination (CIE; Commission Internationale de l’Éclairage) from psychophysical measurements in a group of human observers, and generally *V*(*λ*) can be modeled as a weighted sum of the long-wavelength-sensitive (*L*) and middle-wavelength-sensitive (*M*) cone spectral sensitivities. In 1931, the CIE developed a system of colorimetry to describe color objectively, resulting in the CIE 1931 XYZ system (in which *V*(*λ*) is included as the *ȳ*(*λ*) function).

In 2018, a novel system of metrology was developed by the CIE to quantify the activation of all human photoreceptors with specific relevance to the physiological and behavioral effects of light, incorporating also the melanopsin-containing ipRGCs in addition to the long-wavelength-sensitive (*L*), medium-wavelength-sensitive (*M*) and short-wavelength-sensitive (*S*) cones and the rods. This system proposes a set of five spectral sensitivities to calculate the α-opic (ir)radiance, which is the (ir)radiance for a specific retinal photoreceptor class (S cone, M cone, L cone, rhodopsin and melanopsin-encoded photoreception of ipRGCs). It is possible to convert the α-opic values into a colorimetric system like the previously described CIE 1931 XYZ system.

- **Linearity**: The linearity of a sensor refers to whether the sensors respond to linear changes in incident irradiance in a proportional manner. Generally, it is possible to take measurements of spectral sensitivity and linearity in the same set of measurements, if the measurement system allows for both changes in wavelength of monochromatic light and its excitant irradiance.
- **Fatigue**: Fatigue refers to any unpredicted responses in the sensor following prolonged exposure to light. This has so far, at least to our knowledge, not been characterized in wearable light sensors.
- **Dependence on temperature and humidity**: Depending on how the sensors are designed and driven, they may be sensitive to temperature and humidity. Therefore, making generalizable measurements requires establishing that the sensor response is independent of temperature and/or humidity, or to derive correction factors that are temperature and humidity-dependent. This aspect is even more relevant in the context of wearables.
- **Response to modulated light**: Sensors may respond differently to temporally varying or modulated light. The temporal response of the sensor needs to be characterized.
- **Resolution**: The resolution and minimum resolvable light step of the sensors must be characterized.

Taken together, the verification process and the data generated in this process is often opaque in the context of BioMeTs, and certainly so for wearable personal light sensors. This article provides recommendations for the evaluation of light sensors, especially when employed in clinical research settings. While it may not be straightforward to perform these characterizations, the V3 framework and its explicit call upon verification of BioMeTs for clinical research will support the development of a consensus and/or community-driven standards and guidelines.

### Analytical validation of light dosimeters

The analytical validation of light dosimeters refers to the validation of light measurements under well-controlled lighting scenarios made with the dosimeters (Figure 2B). This is most convenient under laboratory conditions, where lighting conditions can be held constant and accurately calibrated using high-specification research-grade stationary spectrometers, hyper-spectral cameras or luminance cameras. If a BioMeTs under investigation generates light measurements that inaccurately represents light exposure under these parametric conditions, it is very unlikely that it can also support measurements in the context of clinical trials in real-world settings. Depending on the types of sensor channels contained in a dosimeter, there are obvious ways to check the (ecological) validity of the measurements. For example, if a dosimeter has a UV channel, then under typical indoors without any daylight condition, the UV irradiance should be minimal. However, in outdoor light conditions, measurements might be heavily affected. A relatively weak but occasionally useful form of analytical validation is the cross-calibration of BioMeTs to be used in a study. This could involve subjecting the light dosimeters to the same set of standard conditions – e.g., an overcast sky^84^ – and then generating a correction factor to at least make devices used within a study to yield comparable data.

### Clinical validation of light dosimeters

The clinical validation of light dosimeters demonstrates their usability in describing, predicting or explaining health or clinically relevant outcomes (Figure 2C). As described above, there are various studies that have described naturalistic light exposure as a function of patient group, geographical location and season, and/or associated light exposure with health outcomes. Moreover, light dosimeters have been used to predict health-related outcome parameters in both clinical and non-clinical populations, rendering mixed findings (see, e.g., Ref.^52^). The degree to which light dosimeters are useful for clinical contexts requires further research over long periods of time, using sensors that have been adequately assessed from verification and analytical validation perspectives. A key consideration is that the criteria used in characterizing a light dosimeter may differ between different types of trials and outcomes used.

### Open questions in digital dosimetry

While the V3 framework provides a framework for considering the key technical and technological challenges for personalized sensor light dosimetry, there are additional questions that require theoretical and practical clarification.

### To what extent is corneal light exposure a physiologically relevant quantity?

As stated earlier, retinal irradiance cannot be practically measured. Even when an observer is stationary and the scene constant, the light effectively reaching the eye and retina will be modulated in time by individual-level characteristics (such as head movements, eyelid closure, facial features, and pupil size^18,85^). Using some assumptions, e.g., a parametric model of pupil size as a function of corneal irradiance,^86,87^ the retinal irradiance could be reconstructed from corneal irradiance, but not in a spectrally selective way. In the limit, it is possible to conceive of two spatially very distinct scenarios yielding the same spatially integrated corneal irradiance: One very bright point light source in an otherwise dark field and one homogenous field. Importantly, the physiological effect of light varies with the region of the illuminated retina.^88–93^ At present, spatially resolved measurements are not generally available for wearable dosimetry to incorporate these effects.

Additionally, even if it is possible to estimate the spatially resolved and temporally defined pattern of α-opic radiances at the level of the retina, it is the photoreceptor signals that are relayed to targets in the brain that underlie the physiological effects of light. These may be subject to non-linearities, and may also be combined. For example, the ipRGCs receive input from the cones and rods,^94,95^ suggesting a pathway for integration of photoreceptor signals at the level of the retina already. For practical purposes, even if there is a biological capacity for the cones and rods to contribute to the physiological effects of light, there is converging evidence that melanopic quantities may be sufficient to predict them under most conditions.^96–100^

### What is the usability and acceptability of digital dosimeters?

Different form factors for dosimeters place different demands on participants. Whether or not a portable sensor is usable will modify the extent to which it is fit-for-purpose in the field. While feasibility and acceptability concerns are typically considered during the planning phase of interventional clinical trials, it is critical to assess the usability, acceptability and equity of BioMeTs to be used in digital health and medicine. It is important to mention that there is typically a tradeoff between usability of light sensors and their accuracy in terms of approximation of corneal light exposure. For instance, sensors worn at the wrist might be preferred by users over the use of sensors mounted on a pair of glasses, but at the same time these sensors might render less accurate proxies of retinal light exposure as they capture light in a different plane and are more likely to be covered. Yet, usability is crucial for users’ adherence to the required (long-term) measurement protocol. Missing data, especially when systematic in specific contexts (such as when being in company of others and/or when spending time outdoors), might inhibit the collection of representative light exposure profiles in everyday-life settings.

### Which metrics should be used?

An important consideration is that there is currently no standard set of metrics for summarizing time series data obtained with light sensors. This is particularly relevant for field assessments, with substantial inter- and intra-individual variations in light exposure profiles as a function of season, time of day and weather conditions and users’ location (e.g., indoors or outdoors) and behavior.^101^ The lack of a standard set of metrics to quantify light exposure patterns challenges comparison across studies.

Moreover, it is not clear whether reported significant associations in the literature arose from a principled process, or whether parameters were tweaked until a significant association was found without statistical penalty. A potential solution is a multiverse approach,^102^ preferably employed in a large data set, where a set of plausible choices of analytic parameters are systematically investigated and the robustness of a specific association is assessed. For studies reporting null findings, it could also be the case that the used aggregation missed important features of the light exposure profile that are particularly determinant in light-induced moderations in the outcome parameter.

### How comparable are data from different groups and populations?

Portable sensor dosimeters can generate large amounts of data in different geographical locations or in groups with different demographic make-up. The development of common frameworks for storing and processing BioMeTs data will be a key in facilitating “big data” analyses of light dosimetry, and other types of data collected via digital sensors.^103–105^

#### How to handle non-wear periods and quality control?

The assessment of rest-activity cycles with actigraphy is often paired with the use of a sleep diary,^106–111^ sometimes indicating non-wear periods which can then be masked in later analysis steps. For light dosimetry, there are currently no standard instruments for capturing non-wear periods. In cases when the light-logging is integrated in a wrist-worn device also using actigraphy, the non-wear periods indicated in a sleep diary may be used for determining the periods on non-wear.

Quality control in general is an unsolved problem in light dosimetry, as there is no objective method for determining the congruency of corneal light exposure with measured light exposure. For example, sleeves may cover wrist-worn devices, and scarves may cover light dosimeters attached near the neck of a participant. A standard method of assessing the amount of invalid data needs to be developed.

## Conclusion

Monitoring personalized light exposure through digital sensors is a key growing area in a variety of applications, ranging from its inclusion in associational studies, to light exposure being used as a medical intervention to treat, monitor or diagnose multiple conditions, or as a health-related outcome, moderator, or mediator in clinical trials. While there are now various light dosimeters available both for research use and commercially, it is not clear how to validate and verify them, and standard frameworks for the characterization of these light dosimeters do not necessarily apply. This article described the verification, analytic validation, and clinical validation (V3) framework to digital light-dosimetry sensors and highlighted ongoing challenges for portable digital dosimetry.

## References

[1] Blume C Garbazza C Spitschan M. Effects of light on human circadian rhythms, sleep and mood. Somnologie (Berl) 2019; 23: 147–156. 31534436 10.1007/s11818-019-00215-x PMC6751071

[2] Vetter C Morgan Pattison P William Houser K, et al. A review of human physiological responses to light: implications for the development of integrative lighting solutions. Leukos 2021; 18(3): 387–328.

[3] Schwartz WJ Klerman EB. Circadian neurobiology and the physiologic regulation of sleep and wakefulness. Neurol Clin 2019; 37: 475–486. 31256784 10.1016/j.ncl.2019.03.001 PMC6604835

[4] Haspel JA Anafi R Brown MK, et al. Perfect timing: circadian rhythms, sleep, and immunity – an NIH workshop summary. JCI Insight 2020; 5. 10.1172/jci.insight.131487 PMC7030790 31941836

[5] Poggiogalle E Jamshed H Peterson CM. Circadian regulation of glucose, lipid, and energy metabolism in humans. Metabolism 2018; 84: 11–27. 29195759 10.1016/j.metabol.2017.11.017 PMC5995632

[6] Fishbein AB Knutson KL Zee PC. Circadian disruption and human health. J Clin Invest 2021; 131: e148286. 34596053 10.1172/JCI148286 PMC8483747

[7] Vetter C. Circadian disruption: what do we actually mean? Eur J Neurosci 2020; 51: 531–550. 30402904 10.1111/ejn.14255 PMC6504624

[8] Walker WH II Walton JC DeVries AC, et al. Circadian rhythm disruption and mental health. Transl Psychiatry 2020; 10: 28. 32066704 10.1038/s41398-020-0694-0 PMC7026420

[9] Cajochen C. Alerting effects of light. Sleep Med Rev 2007; 11: 453–464. 17936041 10.1016/j.smrv.2007.07.009

[10] Hanifin JP Brainard GC. Photoreception for circadian, neuroendocrine, and neurobehavioral regulation. J Physiol Anthropol 2007; 26: 87–94. 17435349 10.2114/jpa2.26.87

[11] Souman JL Tinga AM te Pas SF, et al. Acute alerting effects of light: a systematic literature review. Behav Brain Res 2018; 337: 228–239. 28912014 10.1016/j.bbr.2017.09.016

[12] Lok R Smolders KCHJ Beersma DGM, et al. Light, alertness, and alerting effects of white light: a literature overview. J Biol Rhythms 2018; 33: 589–601. 30191746 10.1177/0748730418796443 PMC6236641

[13] Brown TM Brainard GC Cajochen C, et al. Recommendations for daytime, evening, and nighttime indoor light exposure to best support physiology, sleep, and wakefulness in healthy adults. PLoS Biol 2022; 20: e3001571. 35298459 10.1371/journal.pbio.3001571 PMC8929548

[14] (UL), U.L., UL DG 24480: Design Guideline for Promoting Circadian Entrainment with Light for Day-Active People. 2019.

[15] WELL. *The WELL Building Standard: Circadian Lighting Design*. 2020.

[16] DIN. DIN SPEC 67600:2013-04. Biologically Effective Illumination – Design guidelines/Biologisch Wirksame Beleuchtung – Planungsempfehlungen. Berlin: Beuth Verlag GmbH, 2013.

[17] Stefani O Cajochen C. Should we re-think regulations and standards for lighting at workplaces? A practice review on existing lighting recommendations. Front Psychiatry 2021; 12: 652161. 34054611 10.3389/fpsyt.2021.652161 PMC8155670

[18] Spitschan M. Time-varying light exposure in chronobiology and sleep research experiments. Front Neurol 2021; 12: 654158. 34335437 10.3389/fneur.2021.654158 PMC8319561

[19] Webler FS Spitschan M Foster RG, et al. What is the ‘spectral diet’ of humans? Curr Opin Behav Sci 2019; 30: 80–86. 31431907 10.1016/j.cobeha.2019.06.006 PMC6701986

[20] Hartmeyer SL Webler FS Andersen M. Towards a framework for light-dosimetry studies: Methodological considerations. Light Res Technol 2022.

[21] Webler FS Chinazzo G Andersen M. Towards a wearable sensor for spectrally-resolved personal light monitoring. J Phys Conf Ser 2021; 2042.

[22] Bierman A Klein TR Rea MS. The daysimeter: a device for measuring optical radiation as a stimulus for the human circadian system. Meas Sci Technol 2005; 16: 2292–2299.

[23] Hubalek S. LuxBlick: Messung der täglichen Lichtexposition zur Beurteilung der nicht-visuellen Lichtwirkungen über das Auge. Zürich: Eidgenössische Technische Hochschule ETH Zürich, 2007.

[24] Stampfli JR Schrader B di Battista C, et al. The light-dosimeter – a new device to help advance research on the non-visual responses to light. Proceedings of the Conference CIE 2021, 2021: CIE. 10.1177/14771535221147140 PMC10353031 37469656

[25] Price LL Lyachev A Khazova M. Optical performance characterization of light-logging actigraphy dosimeters. J Opt Soc Am A Opt Image Sci Vis 2017; 34: 545–557. 28375324 10.1364/JOSAA.34.000545

[26] Okudaira N Kripke DF Webster JB. Naturalistic studies of human light exposure. Am J Physiol 1983; 245: R613–R615. 6624956 10.1152/ajpregu.1983.245.4.R613

[27] Duncan DD Schneider W West KJ, et al. The development of personal dosimeters for use in the visible and ultraviolet wavelengths regions. The salisbury eye evaluation team. Photochem Photobiol 1995; 62: 94–100. 7638275 10.1111/j.1751-1097.1995.tb05244.x

[28] Kwon K Heo SY Yoo I, et al. Miniaturized, light-adaptive, wireless dosimeters autonomously monitor exposure to electromagnetic radiation. Sci Adv 2019; 5: eaay2462. 31853499 10.1126/sciadv.aay2462 PMC6910837

[29] Cao Y Lan W Wen L, et al. An effectiveness study of a wearable device (Clouclip) intervention in unhealthy visual behaviors among school-age children: a pilot study. Medicine (Baltimore) 2020; 99: e17992. 31914011 10.1097/MD.0000000000017992 PMC6959882

[30] Wen L Cheng Q Cao Y, et al. The clouclip, a wearable device for measuring near-work and outdoor time: validation and comparison of objective measures with questionnaire estimates. Acta Ophthalmol 2021; 99: e1222–e1235. 33729708 10.1111/aos.14785

[31] Aarts MPJ van Duijnhoven J Aries MBC, et al. Performance of personally worn dosimeters to study non-image forming effects of light: assessment methods. Build Environ 2017; 117: 60–72.

[32] van Hoof J Westerlaken AC Aarts MPJ, et al. Light therapy: methodological issues from an engineering perspective. Technol Health Care 2012; 20: 11–23. 22297710 10.3233/THC-2011-0650

[33] Jardim AC Pawley MDM Cheeseman JF, et al. Validating the use of wrist-level light monitoring for in-hospital circadian studies. Chronobiol Int 2011; 28: 834–840. 21936617 10.3109/07420528.2011.611603

[34] Figueiro MG Hamner R Bierman A, et al. Comparisons of three practical field devices used to measure personal light exposures and activity levels. Light Res Technol 2013; 45: 421–434. 24443644 10.1177/1477153512450453 PMC3892948

[35] Price LLA Khazova M O’Hagan JB. Performance assessment of commercial circadian personal exposure devices. Light Res Technol 2012; 44: 17–26.

[36] Bhandari KR Mirhajianmoghadam H Ostrin LA. Wearable sensors for measurement of viewing behavior, light exposure sleep. Sensors (Basel) 2021; 21. 10.3390/s21217096 PMC8587946 34770402

[37] Stone JE McGlashan EM Facer-Childs ER, et al. Accuracy of the GENEActiv device for measuring light exposure in sleep and circadian research. Clocks Sleep 2020; 2: 143–152. 33089197 10.3390/clockssleep2020012 PMC7445795

[38] Joyce DS Zele AJ Feigl B, et al. The accuracy of artificial and natural light measurements by actigraphs. J Sleep Res 2020; 29: e12963. 31860938 10.1111/jsr.12963

[39] Howell CM McCullough SJ Doyle L, et al. Reliability and validity of the Actiwatch and Clouclip for measuring illumination in real-world conditions. Ophthalmic Physiol Opt 2021; 41: 1048–1059. 34387902 10.1111/opo.12860

[40] Cao D Barrionuevo PA. Estimating photoreceptor excitations from spectral outputs of a personal light exposure measurement device. Chronobiol Int 2015; 32: 270–280. 25290040 10.3109/07420528.2014.966269 PMC4355054

[41] Spitschan M Stefani O Blattner P, et al. How to report light exposure in human chronobiology and sleep research experiments. Clocks Sleep 2019; 1: 280–289. 31281903 10.3390/clockssleep1030024 PMC6609447

[42] Campbell SS Kripke DF Gillin JC, et al. Exposure to light in healthy elderly subjects and Alzheimer’s patients. Physiol Behav 1988; 42: 141–144. 3368532 10.1016/0031-9384(88)90289-2

[43] Savides TJ Messin S Senger C, et al. Natural light exposure of young adults. Physiol Behav 1986; 38: 571–574. 3823171 10.1016/0031-9384(86)90427-0

[44] Jean-Louis G Kripke DF Ancoli-Israel S, et al. Sleep duration, illumination, and activity patterns in a population sample: effects of gender and ethnicity. Biol Psychiatry 2000; 47: 921–927. 10807965 10.1016/s0006-3223(99)00169-9

[45] Loving RT Kripke DF. Daily light exposure among psychiatric inpatients. J Psychosoc Nurs Ment Health Serv 1992; 30: 15–19. 10.3928/0279-3695-19921101-06 1494143

[46] Hebert M Dumont M Paquet J. Seasonal and diurnal patterns of human illumination under natural conditions. Chronobiol Int 1998; 15: 59–70. 9493715 10.3109/07420529808998670

[47] Oren DA Moul DE Schwartz PJ, et al. Exposure to ambient light in patients with winter seasonal affective disorder. Am J Psychiatry 1994; 151: 591–593. 8147459 10.1176/ajp.151.4.591

[48] Wams EJ Woelders T Marring I, et al. Linking light exposure and subsequent sleep: a field polysomnography study in humans. Sleep 2017; 40:zsx165. 10.1093/sleep/zsx165 PMC5806586 29040758

[49] Estevan I Tassino B Vetter C, et al. Bidirectional association between light exposure and sleep in adolescents. J Sleep Res 2022; 31(2): e13501. 34608708 10.1111/jsr.13501

[50] Reid KJ Santostasi G Baron KG, et al. Timing and intensity of light correlate with body weight in adults. PLoS One 2014; 9: e92251. 24694994 10.1371/journal.pone.0092251 PMC3973603

[51] Hubalek S Brink M Schierz C. Office workers’ daily exposure to light and its influence on sleep quality and mood. Light Res Technol 2010; 42: 33–50.

[52] Smolders KCHJ de Kort YAW van den Berg SM. Daytime light exposure and feelings of vitality: results of a field study during regular weekdays. J Environ Psychol 2013; 36: 270–279.

[53] Bohmer MN Hamers PCM Bindels PJE, et al. Are we still in the dark? A systematic review on personal daily light exposure, sleep-wake rhythm, and mood in healthy adults from the general population. Sleep Health 2021; 7: 610–630. 34420891 10.1016/j.sleh.2021.06.001

[54] Dumont M Beaulieu C. Light exposure in the natural environment: relevance to mood and sleep disorders. Sleep Med 2007; 8: 557–565. 17383230 10.1016/j.sleep.2006.11.008

[55] Stothard ER McHill AW Depner CM, et al. Circadian entrainment to the natural light-dark cycle across seasons and the weekend. Curr Biol 2017; 27: 508–513. 28162893 10.1016/j.cub.2016.12.041 PMC5335920

[56] Sharkey KM Carskadon MA Figueiro MG, et al. Effects of an advanced sleep schedule and morning short wavelength light exposure on circadian phase in young adults with late sleep schedules. Sleep Med 2011; 12: 685–692. 21704557 10.1016/j.sleep.2011.01.016 PMC3145013

[57] Figueiro MG Steverson B Heerwagen J, et al. The impact of daytime light exposures on sleep and mood in office workers. Sleep Health 2017; 3: 204–215. 28526259 10.1016/j.sleh.2017.03.005

[58] Woelders T Beersma DGM Gordijn MCM, et al. Daily light exposure patterns reveal phase and period of the human circadian clock. J Biol Rhythms 2017; 32: 274–286. 28452285 10.1177/0748730417696787 PMC5476188

[59] Facer-Childs ER Middleton B Skene DJ, et al. Resetting the late timing of ‘night owls’ has a positive impact on mental health and performance. Sleep Med 2019; 60: 236–247. 31202686 10.1016/j.sleep.2019.05.001

[60] Holtmann M Mokros L Kirschbaum-Lesch I, et al. Adolescent depression: study protocol for a randomized, controlled, double-blind multicenter parallel group trial of bright light therapy in a naturalistic inpatient setting (DeLight). Trials 2018; 19: 568. 30340625 10.1186/s13063-018-2949-0 PMC6194631

[61] Swanson LM Burgess HJ Zollars J, et al. An open-label pilot study of a home wearable light therapy device for postpartum depression. Arch Womens Ment Health 2018; 21: 583–586. 29603017 10.1007/s00737-018-0836-z PMC6234841

[62] Starreveld DEJ Daniels LA Valdimarsdottir HB, et al. Light therapy as a treatment of cancer-related fatigue in (non-)Hodgkin lymphoma survivors (SPARKLE trial): study protocol of a multicenter randomized controlled trial. BMC Cancer 2018; 18: 880. 30200906 10.1186/s12885-018-4746-2 PMC6131816

[63] Neikrug AB Rissling M Trofimenko V, et al. Bright light therapy protects women from circadian rhythm desynchronization during chemotherapy for breast cancer. Behav Sleep Med 2012; 10: 202–216. 22742438 10.1080/15402002.2011.634940

[64] Ancoli-Israel S Rissling M Neikrug A, et al. Light treatment prevents fatigue in women undergoing chemotherapy for breast cancer. Support Care Cancer 2012; 20: 1211–1219. 21660669 10.1007/s00520-011-1203-z PMC3192914

[65] Riemersma-van der Lek RF Swaab DF Twisk J, et al. Effect of bright light and melatonin on cognitive and noncognitive function in elderly residents of group care facilities: a randomized controlled trial. JAMA 2008; 299: 2642–2655. 18544724 10.1001/jama.299.22.2642

[66] Cole RJ Smith JS Alcal YC, et al. Bright-light mask treatment of delayed sleep phase syndrome. J Biol Rhythms 2002; 17: 89–101. 11837952 10.1177/074873002129002366

[67] van der Lely S Frey S Garbazza C, et al. Blue blocker glasses as a countermeasure for alerting effects of evening light-emitting diode screen exposure in male teenagers. J Adolesc Health 2015; 56: 113–119. 25287985 10.1016/j.jadohealth.2014.08.002

[68] Lunn RM Blask DE Coogan AN, et al. Health consequences of electric lighting practices in the modern world: a report on the National Toxicology Program’s workshop on shift work at night, artificial light at night, and circadian disruption. Sci Total Environ 2017; 607–608: 1073–1084. 10.1016/j.scitotenv.2017.07.056 PMC5587396 28724246

[69] Obayashi K Yamagami Y Kurumatani N, et al. Bedroom lighting environment and incident diabetes mellitus: a longitudinal study of the HEIJO-KYO cohort. Sleep Med 2020; 65: 1–3. 31704511 10.1016/j.sleep.2019.07.006

[70] Davis S Mirick DK Stevens RG. Night shift work, light at night, and risk of breast cancer. J Natl Cancer Inst 2001; 93: 1557–1562. 11604479 10.1093/jnci/93.20.1557

[71] Kyba CCM Spitschan M. Comment on ‘Domestic light at night and breast cancer risk: a prospective analysis of 105000 UK women in the generations study’. Br J Cancer 2019; 120: 276–277. 30584260 10.1038/s41416-018-0203-x PMC6342976

[72] Johns LE Jones ME Schoemaker MJ, et al. Domestic light at night and breast cancer risk: a prospective analysis of 105 000 UK women in the generations study. Br J Cancer 2018; 118: 600–606. 29360812 10.1038/bjc.2017.359 PMC5830585

[73] Obayashi K Saeki K Kurumatani N. Bedroom light exposure at night and the incidence of depressive symptoms: a longitudinal study of the HEIJO-KYO cohort. Am J Epidemiol 2018; 187: 427–434. 28992236 10.1093/aje/kwx290

[74] Koo YS Song J-Y Joo E-Y, et al. Outdoor artificial light at night, obesity, and sleep health: cross-sectional analysis in the KoGES study. Chronobiol Int 2016; 33: 301–314. 26950542 10.3109/07420528.2016.1143480

[75] McFadden E Jones ME Schoemaker MJ, et al. The relationship between obesity and exposure to light at night: cross-sectional analyses of over 100,000 women in the breakthrough generations study. Am J Epidemiol 2014; 180: 245–250. 24875371 10.1093/aje/kwu117

[76] Park YM White AJ Jackson CL, et al. Association of exposure to artificial light at night while sleeping with risk of obesity in women. JAMA Intern Med 2019; 179: 1061–1071. 31180469 10.1001/jamainternmed.2019.0571 PMC6563591

[77] Pattinson CL Allan AC Staton SL, et al. Environmental light exposure is associated with increased body mass in children. PLoS One 2016; 11: e0143578. 26735299 10.1371/journal.pone.0143578 PMC4711797

[78] Hebert M, et al. The effects of prior light history on the suppression of melatonin by light in humans. J Pineal Res 2002; 33: 198–203. 12390501 10.1034/j.1600-079x.2002.01885.x PMC3925650

[79] Chang AM Scheer FA Czeisler CA. The human circadian system adapts to prior photic history. J Physiol 2011; 589: 1095–1102. 21224217 10.1113/jphysiol.2010.201194 PMC3060589

[80] Smith KA Schoen MW Czeisler CA. Adaptation of human pineal melatonin suppression by recent photic history. J Clin Endocrinol Metab 2004; 89: 3610–3614. 15240654 10.1210/jc.2003-032100

[81] Te Kulve M Schlangen LJM van Marken Lichtenbelt WD. Early evening light mitigates sleep compromising physiological and alerting responses to subsequent late evening light. Sci Rep 2019; 9: 16064. 31690740 10.1038/s41598-019-52352-w PMC6831674

[82] Goldsack JC Coravos A Bakker JP, et al. Verification, analytical validation, and clinical validation (V3): the foundation of determining fit-for-purpose for biometric monitoring technologies (BioMeTs). NPJ Digit Med 2020; 3: 55. 32337371 10.1038/s41746-020-0260-4 PMC7156507

[83] ISO/CIE. *ISO/CIE 19476:2014: Characterization of the performance of illuminance meters and luminance meters*. 2014.

[84] Markvart J Hansen ÅM Christoffersen J. Comparison and correction of the light sensor output from 48 wearable light exposure devices by using a side-by-side field calibration method. Leukos 2015; 11: 155–171.

[85] Sliney DH. Exposure geometry and spectral environment determine photobiological effects on the human ey. Photochem Photobiol 2005; 81: 483–489. 15755194 10.1562/2005-02-14-RA-439

[86] Watson AB Yellott JI. A unified formula for light-adapted pupil size. J Vis 2012; 12: 12. 10.1167/12.10.12 23012448

[87] Bracke P Van de Putte E Ryckaert WR. Comment concerning the effects of light intensity on melatonin suppression in the review “light modulation of human clocks, wake, and sleep” by A. Prayag, et al. Clocks Sleep 2021; 3: 181–188. 33578834 10.3390/clockssleep3010011 PMC7931082

[88] Rea MS Nagare R Figueiro MG. Relative light sensitivities of four retinal hemi-fields for suppressing the synthesis of melatonin at night. Neurobiol Sleep Circadian Rhythms 2021; 10: 100066. 33997475 10.1016/j.nbscr.2021.100066 PMC8099627

[89] Ruger M Gordijn MCM Beersma DGM, et al. Weak relationships between suppression of melatonin and suppression of sleepiness/fatigue in response to light exposure. J Sleep Res 2005; 14: 221–227. 16120096 10.1111/j.1365-2869.2005.00452.x

[90] Ruger M Gordijn MCM Beersma DGM, et al. Nasal versus temporal illumination of the human retina: effects on core body temperature, melatonin, and circadian phase. J Biol Rhythms 2005; 20: 60–70. 15654071 10.1177/0748730404270539

[91] Glickman G Hanifin JP Rollag MD, et al. Inferior retinal light exposure is more effective than superior retinal exposure in suppressing melatonin in humans. J Biol Rhythms 2003; 18: 71–79. 12568246 10.1177/0748730402239678

[92] Smith JS Kripke DF Elliott JA, et al. Illumination of upper and middle visual fields produces equivalent suppression of melatonin in older volunteers. Chronobiol Int 2002; 19: 883–891. 12405551 10.1081/cbi-120014107

[93] Lasko TA Kripke DF Elliot JA. Melatonin suppression by illumination of upper and lower visual fields. J Biol Rhythms 1999; 14: 122–125. 10194648 10.1177/074873099129000506

[94] Dacey DM Liao H-W Peterson BB, et al. Melanopsin-expressing ganglion cells in primate retina signal colour and irradiance and project to the LGN. Nature 2005; 433: 749–754. 15716953 10.1038/nature03387

[95] Patterson SS Kuchenbecker JA Anderson JR, et al. A color vision circuit for non-image-forming vision in the primate retina. Curr Biol 2020; 30: 1269–1274. e2. 32084404 10.1016/j.cub.2020.01.040 PMC7141953

[96] Brown TM. Melanopic illuminance defines the magnitude of human circadian light responses under a wide range of conditions. J Pineal Res 2020; 69: e12655. 32248548 10.1111/jpi.12655

[97] Prayag AS Najjar RP Gronfier C. Melatonin suppression is exquisitely sensitive to light and primarily driven by melanopsin in humans. J Pineal Res 2019; 66: e12562. 30697806 10.1111/jpi.12562

[98] Nowozin C Wahnschaffe A Rodenbeck A, et al. Applying melanopic lux to measure biological light effects on melatonin suppression and subjective sleepiness. Curr Alzheimer Res 2017; 14: 1042–1052. 28545361 10.2174/1567205014666170523094526

[99] Hommes V Gimenez MC. A revision of existing Karolinska Sleepiness Scale responses to light: a melanopic perspective. Chronobiol Int 2015; 32: 750–756. 26102373 10.3109/07420528.2015.1043012

[100] Gimenez MC Stefani O Cajochen C, et al. Predicting melatonin suppression by light in humans: unifying photoreceptor-based equivalent daylight illuminances, spectral composition, timing and duration of light exposure. J Pineal Res 2022; 72: e12786. 34981572 10.1111/jpi.12786 PMC9285453

[101] Peeters ST Smolders KCHJ de Kort YAW. What you set is (not) what you get: How a light intervention in the field translates to personal light exposure. Build Environ 2020; 185: 107288.

[102] Simonsohn U Simmons JP Nelson LD. Specification curve analysis. Nat Hum Behav 2020; 4: 1208–1214. 32719546 10.1038/s41562-020-0912-z

[103] Spitschan M Santhi N. Individual differences and diversity in human physiological responses to light. EBioMedicine 2022; 75: 103640. 35027334 10.1016/j.ebiom.2021.103640 PMC8808156

[104] Mazzotti DR Haendel MA McMurry JA, et al. Sleep and Circadian Informatics Data Harmonization: A Workshop Report from the Sleep Research Society and Sleep Research Network. Sleep 2022; 45: zsac002. 35030631 10.1093/sleep/zsac002 PMC9189941

[105] Redline S Purcell SM. Sleep and Big Data: harnessing data, technology, and analytics for monitoring sleep and improving diagnostics, prediction, and interventions-an era for Sleep-Omics? Sleep 2021; 44(6): zsab107. 33893509 10.1093/sleep/zsab107 PMC8521745

[106] Acker JG Becker-Carus C Büttner-Teleaga A, et al. The role of actigraphy in sleep medicine. Somnologie 2021; 25: 89–98.

[107] Tetreault E Bélanger M-È Bernier A, et al. Actigraphy data in pediatric research: the role of sleep diaries. Sleep Med 2018; 47: 86–92. 29778919 10.1016/j.sleep.2017.11.1144

[108] Short MA Gradisar M Lack LC, et al. Estimating adolescent sleep patterns: parent reports versus adolescent self-report surveys, sleep diaries, and actigraphy. Nat Sci Sleep 2013; 5: 23–26. 23620690 10.2147/NSS.S38369 PMC3630985

[109] Schoch SF Kurth S Werner H. Actigraphy in sleep research with infants and young children: current practices and future benefits of standardized reporting. J Sleep Res 2021; 30: e13134. 32638500 10.1111/jsr.13134 PMC8244022

[110] Berger AM Wielgus KK Young-McCaughan S, et al. Methodological challenges when using actigraphy in research. J Pain Symptom Manage 2008; 36: 191–199. 18400460 10.1016/j.jpainsymman.2007.10.008 PMC2542506

[111] Ancoli-Israel S Cole R Alessi C, et al. The role of actigraphy in the study of sleep and circadian rhythms. Sleep 2003; 26: 342–392. 12749557 10.1093/sleep/26.3.342

